# Anaemia, Nutrition, and Caregiver Feeding Practices Among Infants Aged 6 to 8 Months in Maputo City, Mozambique (2024)

**DOI:** 10.3390/nu17162585

**Published:** 2025-08-08

**Authors:** Érica Manuel, Francisco Mbofana, Maria do Rosário O. Martins

**Affiliations:** 1 Instituto Superior de Ciências de Saúde—ISCISA, Maputo 1100, Mozambique; mbofana12@gmail.com; 2 Global Health and Tropical Medicine, GHTM, LA-REAL, Instituto de Higiene e Medicina Tropical, IHMT, Universidade NOVA de Lisboa, 1349-008 Lisboa, Portugal

**Keywords:** infant feeding, anaemia, nutritional status, caregivers, knowledge, attitudes and practices, Maputo, Mozambique

## Abstract

**Background/Objectives**: Anaemia and malnutrition remain global public health challenges, particularly affecting low-income countries such as Mozambique, especially during the complementary feeding period. This study aimed to assess caregivers’ knowledge, attitudes, and practices (KAPs) regarding infant feeding and to explore associations with anaemia and nutritional status among infants aged 6 to 8 months in urban Maputo. **Methods**: A cross-sectional baseline survey was conducted in 2024 in two primary health centres. A total of 496 caregiver–child pairs participated. Children underwent anthropometric measurements and haemoglobin testing, and caregivers completed a structured KAP questionnaire. Descriptive statistics were generated, and chi-square tests were used to examine associations between KAP domains and child health outcomes. **Results**: Anaemia was detected in 77.0% of children, with moderate anaemia being most common (48.6%). Eutrophic nutritional status was observed in 73.1% of children, 7.0% had acute malnutrition, and 14.1% were overweight. While 97.3% of caregivers demonstrated positive attitudes, only 52.1% had adequate knowledge and practices. Adequate knowledge was significantly associated with both the absence of anaemia (*p* = 0.001) and eutrophic status (*p* = 0.001). No significant associations were found for attitudes or practices. Caregiver practices were significantly associated with household income, and anaemia was more frequent among children from low-income households. **Conclusions**: Anaemia was highly frequent among 6–8-month-old infants, even among those with normal weight-for-length and knowledgeable caregivers. This paradox highlights the need for early, integrated interventions, such as nutrition education and home fortification with micronutrient powders, and supports the WHO’s recommendation to revisit haemoglobin thresholds in some contexts.

## 1. Introduction

Malnutrition, which includes undernutrition, micronutrient deficiencies, and over-weight, remains a major global health challenge. It contributes to stunting, wasting, impaired immunity, and poor cognitive development and is linked to nearly 45% of all deaths in children under five years of age [1,2,3]. In 2022, 149 million children were stunted, 45 million wasted, and 37 million were overweight [1]. Among the various forms of malnutrition, anaemia, primarily caused by iron deficiency, is one of the most widespread in early childhood affecting 269 million children aged 6–59 months globally in 2019, with the highest prevalence in sub-Saharan Africa (60.2%) [4].

Children aged 6 to 23 months are especially vulnerable to anaemia due to increased iron requirements during this critical stage of growth and development. Anaemia at this stage is associated with poor dietary quality, limited caregiver knowledge increasing risks of infection, developmental delays, and mortality [5,6]. In response, the World Health Organization emphasises this period as a window of opportunity for nutritional interventions, particularly focused on complementary feeding practices and adequate micronutrient intake [7,8]. The timely introduction of iron-rich foods is essential. However, ensuring adequate feeding practices during this period depends not only on food availability but also, crucially, on the knowledge, attitudes, and practices (KAPs) of caregivers.

There is growing evidence that caregivers’ KAPs strongly influence feeding behaviours and, consequently, children’s nutritional outcomes. Studies in low- and middle-income countries have shown that adequate caregiver knowledge is associated with timely introduction of complementary foods, improved dietary diversity, and better iron intake in early childhood. For example, a study in Malaysia found that mothers with higher KAP scores were significantly more likely to practice appropriate infant feeding, including early initiation of breastfeeding and timely complementary feeding [7,9]. Similarly, in a multi-country analysis of over 80,000 infants under six months, strong associations between maternal education, knowledge, and household characteristics with infant underweight and wasting were demonstrated, underscoring the importance of informed caregiving practices for preventing malnutrition in early life [10]. These findings support the notion that strengthening KAP is a crucial strategy to reduce anaemia and undernutrition in infants, particularly in settings where food availability is not the main limiting factor.

In this context, particular attention must be given to children aged 6 to 8 months, a vulnerable sub-group within the broader 6–23-month window. During this stage, iron stores acquired at birth become depleted, and the risk of anaemia increases significantly. Assessing feeding practices and caregiver knowledge at this point is essential to identify risks early and inform appropriate interventions [8,10,11].

In Mozambique, the burden of anaemia is particularly severe. According to the most recent national data, approximately 73% of children under five are anaemic, with prevalence rates of 56% in Maputo Province and 44% in Maputo City. The situation is especially critical among infants aged 6 to 11 months, where anaemia affects up to 89% of children, highlighting a period of heightened vulnerability in early infancy [12]. A study conducted in a quaternary health facility in Maputo further revealed that 62.2% of children aged 6 to 59 months were anaemic, with 30.9% of cases classified as moderate and 7.4% as severe [13]. Additionally, a longitudinal analysis of national survey data demonstrated that childhood anaemia has remained persistently high between 2011 and 2023, particularly affecting children aged 6 to 23 months [14].

To address this situation, Mozambique has implemented nutritional interventions such as the distribution of multiple micronutrient powders (MNPs) to improve dietary quality in young children. However, to date, no evaluation has been conducted to assess the effectiveness of these supplements in reducing anaemia or improving nutritional outcomes.

Despite the availability of health services and food in urban areas such as Maputo City, the high prevalence of anaemia indicates that access alone is insufficient. It is essential that caregivers not only receive adequate counselling but also understand and apply the recommendations. Nutritional messages must be clear, adapted to the local reality, and supported by educational materials that are easy to understand and use. There is a need to strengthen mechanisms for monitoring how complementary feeding is being implemented and whether caregivers are following the guidance in practice.

In urban settings, where food is generally available, the persistence of inadequate feeding practices may reflect gaps in communication, follow-up, or caregiver empowerment. Some families may still face challenges in accessing certain foods, but often the problem lies in the way nutritional messages are delivered or understood. Improvements are needed both in the quality of the information provided and in the systems that support and track caregiver practices.

Despite these alarming figures, many health facilities in Mozambique lack the structural capacity to implement essential nutritional interventions, such as routine iron supplementation, growth monitoring, and caregiver education [15]. In Maputo City, physical access to services is comparatively better than in rural areas. Yet, the continued high prevalence of anaemia in urban settings suggests that access alone is not enough. The quality of counselling, the relevance of the information provided, and caregivers’ ability to apply it effectively are central to achieving real improvements. Although anaemia and malnutrition remain prevalent in Mozambique, there is a lack of studies focusing specifically on the 6–8-month age group, combining nutritional status with an analysis of caregivers’ KAPs, which is essential to guide early interventions. This age group marks a critical inflexion point for prevention. Understanding the factors that shape feeding practices at this early stage is essential for designing tailored interventions. This study aims to assess knowledge, attitudes, and practices concerning child feeding, and estimate the frequency of anaemia and nutritional status in children aged 6–8 months attending two health centres in Maputo, Mozambique, in 2024. We also explore associations between KAPs and children’s health outcomes. These results will be used as a baseline for future nutritional interventions.

## 2. Materials and Methods

### 2.1. Study Design

This is a cross-sectional study with a quantitative approach, conducted in two health centres in Maputo.

### 2.2. Study Setting and Period

Mozambique is a low-income country in southeastern Africa, bordered by Tanzania, Malawi, Zambia, Zimbabwe, South Africa, Eswatini, and the Indian Ocean. It is administratively divided into 11 provinces, three in the north (Cabo Delgado, Nampula, and Niassa), four in the centre (Zambezia, Tete, Manica, and Sofala), and four in the south (Gaza, Inhambane, Maputo Province, and Maputo City). Maputo City, the national capital, and Maputo Province are in the southern region and represent the most urbanised areas of the country. The study was conducted in two urban primary health centres in this region: Matola Health Centre at Maputo Province and 1º de Maio Health Centre at Maputo City. Both are part of the National Health Service and provide maternal and child healthcare, including growth monitoring, immunisations, nutritional counselling, and supplementation.

According to health service records, Matola Health Centre provides care to an average of 1800 children under five each month, of whom approximately 300 are aged between 6 and 24 months. In contrast, 1º de Maio Health Centre serves about 6000 children per month; however, data disaggregated by age for children under two years are not available. Although data were collected in two distinct urban health centres, the present analysis adopts an aggregated approach to describe the baseline scenario.

Data collection occurred between February and July 2024 during routine healthy child consultations which are intended to monitor the growth and development of children aged 0 to 59 months. These consultations focus on preventive actions such as growth monitoring (weighing), immunizations, and nutritional counselling.

### 2.3. Study Population

All children aged 6 to 8 months attending routine healthy child consultations at the selected health centres were screened for eligibility. Those who met the inclusion criteria were invited to participate in the study, following the signing of the informed consent form by their respective caregivers.

The study included both children and their caregivers. All eligible children underwent nutritional assessment, regardless of their nutritional status or presence of anaemia. However, participation in the KAP survey was limited to caregivers of children with a eutrophic nutritional status or mild acute malnutrition.

Caregivers of children presenting with moderate acute malnutrition (MAM: weight-for-height z-score between ≥−3 and <−2 standard deviations [SD]), severe acute malnutrition (SAM: weight-for-height z-score <−3 SD), malaria, oedema, severe anaemia (Hb < 7 g/dL), or undergoing therapeutic supplementation (such as RUTF—Ready-to-Use Therapeutic Food or CSB—Corn–Soy Blend) were excluded from the KAP component.

### 2.4. Variables

#### 2.4.1. Main Outcomes

- Haemoglobin levels, treated as a continuous variable and classified according to World Health Organization (WHO) thresholds [16] as follows:
  (a) No anaemia (Hb ≥ 11.0 g/dL);
  (b) Mild anaemia (10.0–10.9 g/dL);
  (c) Moderate anaemia (7.0–9.9 g/dL);
  (d) Severe anaemia (Hb < 7.0 g/dL).
- Nutritional status, based on weight-for-height z-scores (WHZs) using WHO 2006 Growth Standards [17], was categorised as follows:
  (a) Eutrophic (WHZ ≥ −1);
  (b) Mild acute malnutrition (−2 ≤ WHZ < −1);
  (c) Moderate acute malnutrition (−3 ≤ WHZ < −2);
  (d) Severe acute malnutrition (WHZ < −3);
  (e) At risk of overweight/overweight (WHZ > 2).
- Caregivers’ knowledge, attitudes, and practices regarding infant feeding and micronutrient powder (MNP) use.

(a) Knowledge about Infant Feeding

The variable level of knowledge about infant feeding was calculated using the scores of a set of questions. Each correct response was assigned a one-unit score, and each incorrect or “don’t know” response was scored as zero. The total score was converted into a percentage. Respondents whose score was 60% or more were considered to have adequate knowledge, whereas those scoring less than 60% were considered to have inadequate knowledge.

(b) Attitudes to Infant Feeding

The variable attitudes were constructed by assigning scores to a set of attitude-related questions. Scores were added and converted into a percentage. Respondents with a score of 60% or more were classified as having a positive attitude, while those scoring less than 60% were classified as having a negative attitude.

(c) Practices regarding Infant Feeding

The variable feeding practices was calculated using scores from questions about actual behaviours. The scores were summed and converted into a percentage. Respondents with a score of 60% or more were considered to have appropriate practices, and those scoring below 60% were considered to have inappropriate practices.

#### 2.4.2. Socioeconomic and Demographic Characteristics

- Child-level variables: Age and sex.
- Caregiver-level variables: Level of education and monthly household income.

Other variables

- Information Exposure: Caregivers were asked if they received information on infant feeding and its source.

### 2.5. Instruments

The following standardised instruments were used during data collection:

- Clock scale and portable stadiometer—to measure the children’s weight and height. Each measurement was taken twice, with a maximum allowable variation of 100 g for weight and 0.2 cm for height.
- HemoCue Hb 201+ DM device—used to determine haemoglobin levels using capillary blood samples obtained via finger prick.
- Rapid Diagnostic Test (RDT) for malaria—used to detect Plasmodium spp. in capillary blood samples.
- Direct clinical observation for the identification of nutritional oedema—performed by applying firm thumb pressure on both feet for three seconds to identify bilateral oedema.
- The structured KAP questionnaire—to assess caregivers’ KAPs regarding infant feeding. The instrument was adapted from validated tools applied in African contexts and culturally adjusted for Mozambique [10,14,15]. It comprised 28 questions grouped into the following four thematic sections:
  (a) Sociodemographic characteristics (7 questions), including the child’s age, sex, and number of siblings, as well as the caregiver’s age, education level, marital status, and household income.
  (b) Knowledge of complementary feeding and supplementation (8 questions), covering topics such as the timing of complementary feeding, recommended foods, information sources, and knowledge about MNP.
  (c) Attitudes towards feeding practices and the use of MNPs (5 questions), exploring perceptions and beliefs regarding recommended practices, such as exclusive breastfeeding and appropriate food and supplement introduction.
  (d) Feeding practices and MNP use (8 questions), focusing on actual food introduction, types of food offered, duration of exclusive breastfeeding, and the use of nutritional supplements.

### 2.6. Data Collection Procedures

Data collection was conducted by a team of trained healthcare professionals and project researchers, who carried out face-to-face interviews with caregivers and performed anthropometric and clinical assessments of the children, including malaria testing and haemoglobin measurement. All activities took place in private consultation rooms within the health centres to ensure confidentiality and participant well-being.

Prior to the fieldwork, all team members received standardised training in anthropometric measurement techniques, haemoglobin testing, malaria screening, and administration of the structured KAP questionnaire. A pilot study was conducted at the Matola Health Centre to validate procedures and instruments. The data obtained in the pilot phase were consistent and were therefore incorporated into the main study.

Data collection was structured into the following two phases:

Phase 1—Nutritional and clinical assessment of children

All children aged 6 to 8 months were assessed together with their caregivers. Procedures included weight and height measurements, oedema assessment, haemoglobin testing, and malaria screening using rapid diagnostic tests. Clinical and anthropometric data, along with sociodemographic information, were initially recorded on paper-based forms and later digitised into an electronic database.

Phase 2—Administration of the KAP questionnaire to caregivers

Caregivers of eligible children completed the structured KAP questionnaire in a private setting, ensuring confidentiality and comfort during the interview process. Responses were entered directly into an electronic form using Google Forms.

### 2.7. Sample Size

The estimated sample size was 506 caregiver–child pairs, with 253 from each health centre. Although the present analysis focuses on baseline data, the study is part of a broader pre-/post-intervention design. Therefore, the sample size was calculated based on parameters commonly used in intervention studies. The calculation was performed using the statistical software G*Power, version 3.1.9.7, assuming a power of 80% to detect a mean difference of 200 g in children’s body weight between the two groups corresponding to the two participating health centres, Matola Health Centre and 1º de Maio Health Centre, with a 95% confidence level. An allocation ratio of 1 was adopted, and the estimated standard deviation was 0.80, based on similar studies [18,19,20,21,22].

Sample Size Calculation

To compare two independent means, the following statistical formula was used:

*n* = [2 × (Z_1_-α/2 + Z_1_-^β^)^2^ × σ^2^]/Δ^2^

where

*n* = number of individuals per group;
Z_1_-α/2 = 1.96—critical value corresponding to a 5% significance level;
Z_1_-^β^ = 0.84—critical value corresponding to 80% statistical power;
σ = 0.80—estimated standard deviation (in kilograms);
Δ = 0.20—minimum expected difference between group means (in kilograms).

**Figure 1:**
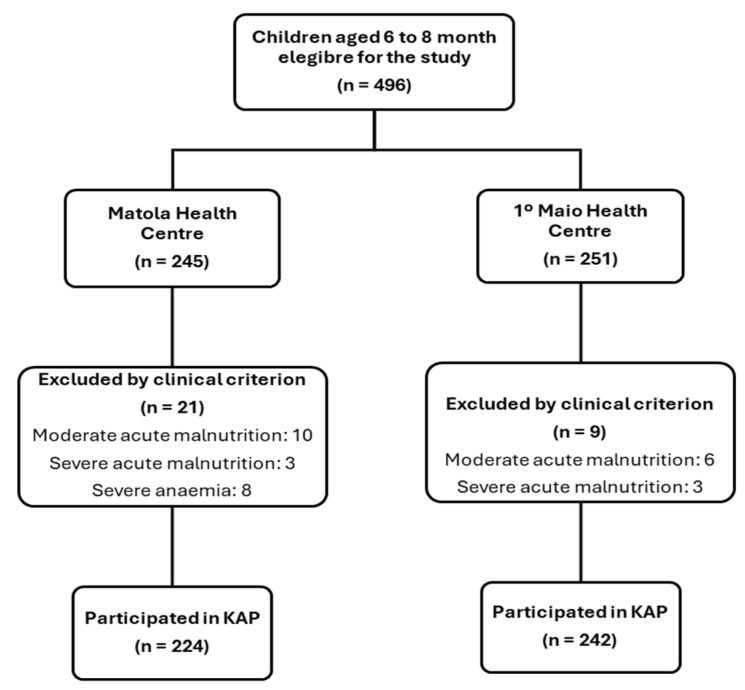
Flowchart of participant selection and inclusion in the study.

Although the estimated sample size was 506 caregiver–child pairs, 10 forms were excluded during data cleaning due to incomplete, inconsistent, or invalid information, resulting in 496 caregiver–child pairs screened during recruitment. Of these, 30 were excluded based on clinical criteria: 16 with moderate acute malnutrition, 6 with severe acute malnutrition, and 8 with severe anaemia (Figure 1). The final sample comprised 466 caregiver–child pairs (224 from Matola Health Centre and 242 from 1º Maio Health Centre) who participated in the KAP survey.

### 2.8. Bias Control

Several strategies were adopted to minimise potential sources of bias. To mitigate information bias, questionnaires were administered by trained professionals, with prior standardisation of questions and the use of language adapted to the caregivers’ sociocultural context. Nevertheless, the possibility of recall bias in responses related to past feeding practices is acknowledged, as is social desirability bias, particularly in questions concerning attitudes and practices. To reduce these effects, anonymity was ensured, and questionnaires were completed in a private setting. However, these strategies are probably not sufficient to eliminate such biases, and this is recognised as a limitation of the present study.

### 2.9. Statistical Analysis

A descriptive analysis was performed for sociodemographic, anthropometric, haemoglobin, and KAP variables. Continuous variables were summarised using measures of central tendency (mean or median) and dispersion (standard deviation or interquartile range), depending on the distribution of data. Categorical variables were presented as absolute and relative frequencies.

The analysis was primarily conducted using aggregated data from the two health centres. Associations between caregivers’ KAPs and child health outcomes (anaemia and nutritional status) were explored using the chi-squared test or Fisher’s exact test, as appropriate.

The Kolmogorov–Smirnov test was used to assess the normality of continuous variables. When normal distribution and homogeneity of variances (verified with Levene’s test) were confirmed, independent samples Student’s *t*-test were used, otherwise a Mann–Whitney U test was applied. Associations between categorical variables, such as household income, KAP indicators, information exposure, and child health outcomes, were examined using Pearson’s chi-squared test (χ^2^). When expected frequencies in contingency tables were below five, Fisher’s exact test was used. A significant level of 5% was adopted. Missing data was excluded from the analyses. Statistical imputation was not performed, as non-response was often considered informative, particularly for knowledge and practice variables. All analyses were conducted using SPSS software, version 27.

## 3. Results

### 3.1. Sociodemographic Characteristics of Children and Caregivers, Nutritional and Anaemia Status of the Children, and Infant Feeding Information

A total of 496 children aged between 6 and 8 months and their respective caregivers participated in the study and were recruited from two urban health centres in Maputo, with analysis conducted in aggregate, given the similar service profiles and population characteristics.

The average age of the children was 6.8 months (SD ± 0.83), and 50.4% were male. Most caregivers (71.1%) had completed secondary education. Regarding household income, 41.9% of families reported earning less than the national minimum wage (8758.00 MZN), as shown in Table 1.

Eutrophy was the most frequently observed nutritional status, present in 73.1% of children. This was followed by overweight (14.1%), mild acute malnutrition (8.3%), moderate acute malnutrition (5.0%), and severe acute malnutrition (1.8%).

Haemoglobin levels were assessed in all participating children. The overall median haemoglobin concentration was 10.0 g/dL (IQR = 1.9), with values ranging from 5.0 to 14.1 g/dL. Anaemia was frequent, affecting 77.0% of the children, with moderate anaemia being the most prevalent form (48.6%), followed by mild (27.4%) and severe anaemia (1.4%).

Exposure to infant feeding information was high among caregivers. In total, 92.0% reported having heard about complementary feeding. Health centres were the most frequently cited source of information (51.9%), followed by both health centres and external sources such as community actors or media (35.0%). Only a small proportion (5.2%) reported receiving information exclusively from outside the health system.

### 3.2. Knowledge, Attitudes, and Practices of Caregivers Regarding Infant Feeding

Adequate knowledge regarding infant feeding, defined as correctly answering at least 60% of the knowledge questions, was identified in 52.1% of caregivers. A positive attitude, defined as scoring at least 60% on attitude-related items reflecting agreement with recommended feeding practices, was observed in 97.3% of participants. Regarding appropriate practices defined as correctly reporting at least 60% of the recommended feeding behaviours, 52.1% of caregivers reported appropriate practices, while 47.9% were classified as having inappropriate practices, as shown in Table 2.

Knowledge regarding the use of liquids as part of complementary feeding was found in 90.1% of caregivers; 90.2% showed knowledge about nutritional education activities, and 76.2% correctly identified the appropriate age for introducing complementary feeding. In contrast, limited knowledge was reported by 87.9% of caregivers concerning the importance of breastmilk, and by 89.9% regarding the use of MNP, as shown in Table 3.

Most caregivers showed favourable attitudes toward infant feeding, particularly regarding participation in nutritional education (96.6%), introducing complementary foods with guidance (95.2%), and exclusive breastfeeding until six months (87.1%), as shown in Table 4.

The most frequent practices were participation in nutritional education (82.6%) and exclusive breastfeeding until six months (73.5%). In contrast, lower frequencies were observed for the timely introduction of complementary feeding (26.5%), offering appropriate foods during the first month (31.1%), and the use of micronutrient powder (2.6%), as shown in Table 5.

### 3.3. Association Between Caregivers’ KAP and Child Health Outcomes

A significant association was found between caregivers’ knowledge and both child anaemia (*p* = 0.001) and nutritional status (*p* = 0.001), as shown in Table 6 and Table 7. Among caregivers with adequate knowledge, 83.8% of children were anaemic. Similarly, malnutrition was observed in 17.5% of children whose caregivers had adequate knowledge. No significant associations were observed between attitudes or practices and either outcome (all *p* > 0.1).

### 3.4. Association Between Household Income, Caregivers’ KAPs, Information Exposure, and Child Health Outcomes

Table 8 presents the association between household income and caregivers’ KAPs. No statistically significant differences were found for knowledge (*p* = 0.847) or attitudes (*p* = 0.242). However, practices were significantly more appropriate among caregivers from households earning above the minimum wage (*p* = 0.001).

Table 9 shows a statistically significant association between household income and the reported source of information on infant feeding (*p* = 0.001). Among caregivers from lower-income households, 76.0% reported receiving information exclusively at health centres, whereas 36.2% of those from higher-income households reported receiving information from both health centres and external sources, such as community actors or media.

Table 10 shows no significant association between household income and children’s nutritional status (*p* = 0.063), although malnutrition appeared slightly more frequent among higher-income households. In contrast, Table 11 shows that anaemia was significantly more frequent among children from households earning up to the minimum wage (*p* = 0.020), highlighting a potential link between socioeconomic vulnerability and iron-deficiency anaemia.

## 4. Discussion

Evidence consistently shows that caregivers’ educational level influences childcare practices, including feeding, hygiene, and health-seeking behaviours, as well as the ability to understand and adopt preventive health messages. This association is recognised by the WHO [1,2,7,8] and the Mozambique Demographic and Health Survey 2022–23 (DHS) [12], and is supported by several studies conducted in similar low- and middle-income settings [5,6,9,18,19,20,23]. In the present study, 41.9% of caregivers had completed secondary or higher education, a considerably higher proportion than that observed in the general population of Maputo City (33%) and Province (20%). This discrepancy may stem from the fact that the DHS includes all household residents, while this study focused on caregivers who actively seek child health services, which may reflect higher exposure to formal education and health information. However, despite relatively high levels of caregiver education, the high prevalence of anaemia observed among children underscores the complex and multifactorial nature of childhood anaemia, which may persist even when caregivers are educated [5,6,24,25].

The socioeconomic conditions of families play a key role in shaping children’s health and nutritional outcomes. While the DHS uses a composite wealth quintile [12], the present study collected self-reported income, revealing that 41.9% of families earn below the national government minimum wage. Although these indicators are not directly comparable, previous studies have similarly used reported income to assess vulnerability in urban and peri-urban settings [18,19,20,23]. Both types of indicators underscore the coexistence of socioeconomically disadvantaged households in areas generally considered more privileged, reinforcing the need for targeted and inclusive interventions. Importantly, we found that household income was significantly associated with caregivers’ practices, but not with knowledge or attitudes. This finding suggests that while information and motivation may be relatively consistent across income groups, the actual ability to implement appropriate practices is constrained by financial resources [5,6,7,24].

The analysis also revealed that household income influenced exposure to information. While most caregivers had heard about complementary feeding and cited health centres as their main source of information, caregivers from higher-income households reported more diverse sources, including community actors and media. This disparity may reflect the greater access that higher-income households have to media platforms (such as television, radio, and the internet) and to community-based sources of information. These structural advantages facilitate exposure to diverse nutrition messages, while lower-income households remain more dependent on health facilities as their primary, and sometimes sole, source of guidance. This finding highlights persistent inequalities in access to health information and underscores the importance of expanding the national Social and Behaviour Change Communication (SBCC) strategies beyond clinical settings to ensure equitable reach and uptake [15,26,27].

Child nutritional status is a sensitive indicator of early-life conditions and caregiving practices [24]. In the present study, the frequency of moderate acute malnutrition was 5.0%, severe acute malnutrition 1.8%, and overweight 14.1% among children aged 6 to 8 months. According to the DHS [12], for children aged 6 to 11 months, the prevalences were 7.0% (moderate), 1.9% (severe), and 3.6% (overweight). Provincial estimates for Maputo City and Province, which refer to children aged 0 to 59 months, report lower rates: in Maputo City, 1.5% (moderate), 0% (severe), and 2.0% (overweight); and in Maputo Province, 1.2%, 0.3%, and 3.0%, respectively. The high frequency of overweight and the presence of acute malnutrition in this sample illustrate a pattern of a double burden of malnutrition [25], possibly related to inappropriate complementary feeding practices [7], and underscore the importance of guiding caregivers toward balanced dietary practices from early infancy.

Anaemia in early childhood remains a critical public health issue in Mozambique, particularly during the transition to complementary feeding [12]. In this study, 77.0% of children aged 6 to 8 months were classified as anaemic, based on the current WHO cutoff of 11.0 g/dL for children aged 6–59 months [16,28]. Although recent studies have proposed lower thresholds, such as 10.5 g/dL, these have not yet been adopted. The WHO acknowledges these proposals and recommends further research to assess the clinical and programmatic implications of revising haemoglobin cutoffs, particularly in high-burden settings [29]. Regardless of the cutoff applied, the high prevalence observed in this study reveals an alarming situation, even in urban areas with relatively better access to health services [12,15]. We also observed a significant association between household income and anaemia, with children from low-income households more frequently affected. This reinforces the argument that poverty-related barriers, such as lack of access to iron-rich foods, poor dietary diversity, and limited supplementation, may have a stronger influence on anaemia than knowledge alone [1,4,13,24,25].

National data from DHS [12] show an even higher prevalence (88.5%) among children aged 6 to 11 months, underscoring the vulnerability of this age group to iron deficiency. Provincial data from the same survey, referring to children aged 0 to 59 months, report anaemia prevalence rates of 43.5% in Maputo City and 56.4% in Maputo Province; figures considerably lower than those observed in the present sample. This discrepancy may partly reflect the broader age range assessed in the DHS; nevertheless, the findings reinforce that anaemia tends to occur with greater intensity during the earliest months of life, even in urban areas with consolidated health infrastructure [12,15,28]. These results highlight that access to health services alone is not sufficient to prevent anaemia, especially when other structural determinants, such as inadequate dietary iron intake, poor adherence to nutritional guidelines, limited coverage of micronutrient supplementation programmes, inadequate WASH (water, sanitation, and hygiene) conditions, and household food insecurity, remain unaddressed [1,4,7,23,24,25,26,27].

Regarding the KAPs of caregivers, although most caregivers in this study demonstrated a positive attitude toward infant feeding (97.3%), only 52.1% reported having adequate knowledge and appropriate practices. These results reveal a substantial gap between caregivers’ attitudes and their actual knowledge and behaviours. Interestingly, this pattern diverges from what is commonly reported in the literature, where knowledge levels tend to be higher than attitudes and practices [9,18,19]. This unexpected discrepancy may reflect limitations in the clarity, accessibility, or applicability of nutritional guidance provided during routine consultations, even in urban settings with better health infrastructure [15,27].

The analysis of specific knowledge items revealed that, while most caregivers correctly identified the appropriate age to introduce complementary feeding (76.2%) and showed strong awareness of the role of liquids and nutritional education (over 90%), important gaps persisted in key areas, such as the importance of breastmilk (with 87.9% showing inadequate knowledge) and the use of MNP, with 89.9% responding incorrectly. These gaps are particularly concerning given that exclusive breastfeeding and adequate supplementation are central strategies for preventing anaemia and malnutrition during infancy [7,8,27].

In terms of practices, 82.6% of caregivers reported participating in nutrition education sessions and 73.5% reported practicing exclusive breastfeeding for up to six months, only 26.5% introduced complementary feeding at the recommended age, and just 2.6% reported using MNP. These results suggest that, despite high exposure to health education delivered by healthcare professionals, the translation of knowledge into behaviour remains limited. This limitation appears to be exacerbated by socioeconomic constraints, particularly among families earning below the minimum wage [5,6,24]. Barriers may include insufficient follow-up, low self-efficacy, or confusion about nutritional recommendations [18,19,20,23].

The feeding patterns observed in this study reflect these limitations. While most children had initiated complementary feeding, the quality and diversity of their diets appear insufficient, as evidenced by the co-occurrence of anaemia and overweight. This aligns with growing evidence that, in urban low- and middle-income settings, the consumption of iron-rich foods remains low, while the exposure to ultra-processed foods is increasing [7,9,20,24]. The high frequency of overweight (14.1%) among children aged 6 to 8 months observed in the present sample, above national and provincial averages [12], suggests the early emergence of the double burden of malnutrition. This reinforces the need for more effective, context-adapted nutrition interventions that go beyond basic counselling and address behavioural, structural, and environmental determinants [7,24,25,26,27].

The association analysis revealed that caregiver knowledge was significantly associated with both child anaemia and nutritional status (*p* = 0.001 for both outcomes). As expected, adequate knowledge was associated with a higher proportion of eutrophic children, suggesting a potential protective role of caregiver knowledge in supporting adequate growth [9,19]. However, an unexpected finding emerged in relation to anaemia: a higher frequency of anaemia was observed among children whose caregivers had adequate knowledge. This paradoxical finding may reflect the complex and multifactorial nature of anaemia, which is not determined by knowledge alone, but also by factors such as poor dietary iron intake, frequent infections, low bioavailability of nutrients, and limited access to micronutrient supplementation [1,4,7,24]. At the same time, although the current WHO cutoff of 11.0 g/dL was applied [16], most children in this study appeared clinically healthy and were cared for by relatively well-informed caregivers. These characteristics align with profiles considered in recent WHO analyses that propose revised haemoglobin thresholds, particularly for healthy populations [29]. This suggests that the high anaemia rates observed may partly reflect limitations in current diagnostic criteria. Similar findings have been reported in other studies, where caregiver knowledge did not always translate into improved nutritional outcomes, particularly in settings marked by poverty, food insecurity, and weak health systems [9,18,23,25]. Altogether, the results reinforce the importance of integrating knowledge-based approaches with actions that address the broader social determinants of health, such as poverty, income, food insecurity, inadequate WASH conditions, and limited access to quality health services, to improve child nutrition outcomes. They also underscore two key challenges: aligning caregiver knowledge with effective practices, and reassessing haemoglobin cutoffs based on population-specific characteristics.

The disconnect between positive attitudes and effective practices highlights the need to strengthen not only the content of nutritional messages but also the methods and contexts through which counselling is delivered. This gap has been documented in other studies, particularly in low- and middle-income settings, where structural, cognitive, and contextual barriers prevent the adoption of recommended feeding practices despite high exposure to health education [9,18,19]. Strategies such as the use of visual aids, reinforcement of messages during each consultation, and adaptation of content to caregivers’ specific profiles may improve retention and adoption of recommended behaviours [20,23,27]. Ensuring that caregivers understand not only what to do, but also why and how to do it, is essential for achieving sustainable improvements in infant feeding.

Finally, although maternal and neonatal data were not collected in this study, maternal anaemia at the time of delivery is a recognised determinant of infant haemoglobin status and should be considered a potential unmeasured confounder. National data from the DHS indicate that 52% of women aged 15–49 years in Mozambique are anaemic, including 43% in Maputo City and 37% in Maputo Province [12], reflecting a substantial burden even in urban settings. Evidence also shows that maternal anaemia may compromise foetal iron stores and contribute to early-onset anaemia during infancy, particularly when exclusive breastfeeding is not accompanied by adequate iron intake or supplementation [30,31,32]. These findings reinforce the need for future studies to adopt a life-course perspective that includes maternal nutritional status before, during, and after pregnancy to better understand intergenerational influences on child health.

## 5. Conclusions

This study highlights the critical issue that, despite high caregiver education and access to health services, anaemia remains highly frequent among infants aged 6–8 months in urban Maputo, even among those with normal nutritional status and caregivers with adequate knowledge. This paradox challenges conventional assumptions and calls for a rethinking of both clinical thresholds and intervention strategies. These findings reinforce the need to address economic disparities and ensure that nutritional education is coupled with strategies that reduce barriers to the implementation of appropriate practices.

Knowledge alone proved insufficient to change practices or protect against poor outcomes, underscoring the urgent need for integrated, practical, and context-sensitive nutrition interventions. Strengthening caregiver support through tailored counselling and home fortification with micronutrient powders may help close the gap between knowledge and practice.

Finally, the findings align with recent WHO recommendations to revisit haemoglobin thresholds in populations where clinical health and anaemia classifications appear misaligned. These insights are essential for shaping early-life interventions that are both evidence-based and contextually relevant, helping to redefine approaches to infant nutrition and anaemia prevention in similar high-burden settings.

Future studies should explore the biological, structural, and social determinants that may mediate this paradox and evaluate the impact of integrated interventions combining caregiver education with micronutrient supplementation on anaemia reduction and child development outcomes.

## 6. Limitations

This study has certain limitations. First, although measures were taken to minimise information bias—such as interviewer training, standardisation of questions, culturally adapted language, and ensuring anonymity—recall bias regarding past feeding practices and social desirability bias in responses on attitudes and practices may still have affected the data.

Second, the study was conducted in two urban health centres in Maputo, which may limit the generalizability of the findings to rural areas or other contexts.

Lastly, although this study focused primarily on caregiver-level determinants, maternal and neonatal data were not collected, limiting our ability to account for early-life influences on infant anaemia, such as maternal haemoglobin or neonatal iron status, which, although beyond the scope of this study, remain important considerations for future research.

## Figures and Tables

**Table 1:** Child and caregiver characteristics, nutritional and anaemia status of the children, and infant feeding information.

Variable	Frequency (*n*)	Percentage (%)
Child characteristics		
Age in months (mean ± SD)	6.81 ± 0.83	
Sex		
Male	250	50.4
Female	246	49.6
Caregiver and household characteristics		
Caregiver’s education		
No formal education	12	2.6
Primary education	89	19.1
Secondary education	332	71.1
Higher education	34	7.3
Household income		
<Minimum wage	195	41.9
=Minimum wage (8758.00 MZN)	121	26.0
>Minimum wage up to 10,000.00 MZN	72	15.5
>10,000.00 MZN	78	16.7
Nutritional status of the children ^1^		
Eutrophic	363	73.1
Mild acute malnutrition	41	8.3
Moderate acute malnutrition	25	5.0
Severe acute malnutrition	9	1.8
Overweight	70	14.1
Anaemia status of the children ^2^		
Haemoglobin (g/dL)		
Median (IQR)	10.0 (1.9)	
Min–Max.	5.0–14.1	
Anaemia classification ^2^		
None (Hb ≥ 11 g/dL)	112	22.6
Mild (10–10.9 g/dL)	136	27.4
Moderate (7–9.9 g/dL)	241	48.6
Severe (<7 g/dL)	7	1.4
Infant feeding information		
Ever heard about complementary feeding		
Yes	429	92.0
No	37	8.0
Source of information		
Only at health centre (HC)	242	51.9
Only outside of HC (media/others)	24	5.2
Both (HC and outside sources)	163	35.0

^1^ Classification based on WHO 2006 weight-for-height Z-scores. ^2^ WHO classification of anaemia in children aged 6–59 months.

**Table 2:** Classification of caregivers according to KAPs regarding infant feeding.

Domain	Classification	Frequency (*n*)	Percentage (%)
Knowledge	Adequate	234	52.1
	Inadequate	215	47.9
	Total	449	100.0
Attitude	Positive	440	97.3
	Negative	12	2.7
	Total	452	100.0
Practice	Appropriate	225	52.1
	Inappropriate	207	47.9
	Total	432	100.0

**Table 3:** Caregivers’ knowledge regarding infant feeding.

Item	Adequate		Inadequate		Total
	*n*	%	*n*	%	*n*
Knowledge about breastmilk	56	12.1	407	87.9	463
Knowledge about solid foods as part of complementary feeding	300	64.9	162	35.1	462
Knowledge about liquids as part of complementary feeding	417	90.1	46	9.9	463
Knowledge about MNP	47	10.1	419	89.9	466
Knowledge about nutritional education activities	412	90.2	45	9.8	457
Knowledge about the age at which to introduce complementary feeding	352	76.2	110	23.8	462

**Table 4:** Caregivers’ attitudes regarding infant feeding.

Attitude Item	Positive		Negative		Total
	*n*	%	*n*	%	*n*
Attitude to exclusive breastfeeding until 6 months	405	87.1	60	12.9	465
Attitude to introducing complementary foods with guidance	441	95.2	22	4.8	463
Attitude to offering water or food before 6 months	196	81.3	45	18.7	241
Attitude to offering MNP to the child	386	83.7	75	16.3	461
Attitude to participation in nutritional education	449	96.6	16	3.4	465

**Table 5:** Caregivers’ practices regarding infant feeding.

Item	Appropriate		Inappropriate		Total
	*n*	%	*n*	%	*n*
Appropriate age for introducing complementary feeding	123	26.5	342	73.5	465
Introduction of complementary feeding with guidance	445	100.0	0	0.0	445
Offering appropriate foods in the first month	141	31.1	313	68.9	454
Exclusive breastfeeding until 6 months	342	73.5	123	26.5	465
Participation in nutritional education	380	82.6	80	17.4	460
Offering of MNP	12	2.6	452	97.4	464

**Table 6:** Association between caregivers’ KAP and anaemia status in children.

Domain	KAP Classification	Anaemia		Total (*n*)	*p*-Value
		Yes (*n*, %)	No (*n*, %)		
Knowledge	Inadequate	149 (69.3%)	66 (30.7%)	215	0.001
	Adequate	196 (83.8%)	38 (16.2%)	234	
Attitude	Negative	11 (91.7%)	1 (8.3%)	12	0.358
	Positive	336 (76,4%)	104 (23.6%)	440	
Practice	Inappropriate	168 (81.2%)	39 (18.8%)	207	0.124
	Appropriate	164 (72.9%)	61 (27.1%)	225	

Note: Significance level: 5%. Pearson’s chi-square test was applied to all comparisons.

**Table 7:** Association between caregivers’ KAPs and nutritional status in children.

Domain	KAP Classification	Malnutrition		Total (*n*)	*p*-Value
		Yes (*n*, %)	No (*n*, %)		
Knowledge	Inadequate	61 (28.4%)	154 (71.6%)	215	0.001
	Adequate	41 (17.5%)	193 (82.5%)	234	
Attitude	Negative	1 (8.3%)	11 (91.7%)	12	0.417
	Positive	104 (23.6%)	336 (76.4%)	440	
Practice	Inappropriate	47 (22.7%)	160 (77.3%)	207	0.734
	Appropriate	47 (20.9%)	178 (79.1%)	225	

Note: Significance level: 5%. Pearson’s chi-square test was applied to all comparisons.

**Table 8:** Association between caregivers’ KAPs and household income.

**Knowledge**				
Income	**Adequate**	**Inadequate**	**Total**	***p*-Value**
>Minimum Wage	90 (54.5%)	75 (45.5%)	165	0.847
≤Minimum Wage	161 (53.4%)	140 (46.5%)	301	
**Attitude**				
Income	**Positive**	**Negative**	**Total**	***p*-Value**
>Minimum Wage	163 (98.8%)	2 (1.2%)	165	0.242
≤Minimum Wage	291 (96.7%)	10 (3.3%)	301	
**Practice**				
Income	**Appropriate**	**Inappropriate**	**Total**	***p*-Value**
>Minimum Wage	110 (66.7%)	55 (33.3%)	165	0.001
≤Minimum Wage	149 (49.5%)	152 (50.5%)	301	

Note: Significance level: 5%. Pearson’s chi-square test was applied to all comparisons.

**Table 9:** Association between sources of information and household income.

Source of Information	≤Minimum Wage *n* (%)	>Minimum Wage *n* (%)	Total (*n*)	*p*-Value
Only at health centres	184 (76.0%)	58 (24.0%)	242	
Only outside health centres	13 (54.2%)	11 (45.8%)	24	
Both (HCs and outside sources)	104 (63.8%)	59 (36.2%)	163	0.001

Note: Significance level: 5%. Pearson’s chi-square test was applied to all comparisons.

**Table 10:** Association between household income and nutritional status in children.

Household Income	Malnutrition		Total (*n*)	*p*-Value
	Yes (*n*, %)	No (*n*, %)		
≤Minimum Wage	65 (20.6%)	251 (79.4%)	316	
>Minimum Wage	43 (28.7%)	107 (71.3%)	150	0.063

Note: Significance level: 5%. Pearson’s chi-square test was applied to all comparisons.

**Table 11:** Association between household income and anaemia status in children.

Household Income	Anaemia		Total (*n*)	*p*-Value
	Yes (*n*, %)	No (*n*, %)		
≤Minimum Wage	253 (80.1%)	63 (19.9%)	316	
>Minimum Wage	106 (70.7%)	44 (29.3%)	150	0.020

Note: Significance level: 5%. Pearson’s chi-square test was applied to all comparisons.

## Data Availability

The data used in this study are not publicly available in a repository but may be made available upon reasonable request to the corresponding author, for the purpose of scientific verification or further research. The study protocol is available for consultation upon request from the corresponding author.

## Abbreviations

The following abbreviations are used in this manuscript:

HB	Haemoglobin
HC	Health Centre
KAP	Knowledge, Attitudes, and Practices
MNP	Multiple Micronutrient Powders
MAM	Moderate Acute Malnutrition
SAM	Severe Acute Malnutrition
INE	Instituto Nacional de Estatística
MISAU	Ministério da Saúde de Moçambique
IOF	Inquérito sobre Orçamento Familiar
WHO	World Health Organization
DHS	Demographic and Health Survey
SD	Standard Deviation
CI	Confidence Interval
